# Exploring Functional Impairment in Light of Prolonged Grief Disorder: A Prospective, Population-Based Cohort Study

**DOI:** 10.3389/fpsyt.2020.537674

**Published:** 2020-12-09

**Authors:** Mette Kjaergaard Nielsen, Kaj Sparle Christensen, Mette Asbjoern Neergaard, Pernille Envold Bidstrup, Mai-Britt Guldin

**Affiliations:** ^1^Research Unit for General Practice, Aarhus, Denmark; ^2^Department of Public Health, Aarhus University, Aarhus, Denmark; ^3^Palliative Care Unit, Department of Oncology, Aarhus University Hospital, Aarhus, Denmark; ^4^Psychological Aspects of Cancer, Danish Cancer Society Research Center, Copenhagen, Denmark; ^5^Department of Psychology, University of Copenhagen, Copenhagen, Denmark

**Keywords:** functional impairment, bereavement, grief, family caregivers, Prolonged Grief Disorder (PGD), SF-36 = 36-item short form health survey, prolonged grief diagnostic criteria

## Abstract

**Background:** Functional impairment is essential in the diagnostic criteria for prolonged grief disorder (PGD) in the ICD-11. It refers to the negative impact on everyday life, including inability to maintain the usual level of functioning. We aimed to assess the extent of functional impairment, emotion-related role limitation, and impaired social functioning before and after bereavement, and to explore associations with PGD, as measured by the 13-item Prolonged Grief Scale (PGD_PG13_).

**Method:** Relatives of terminally ill patients (*n* = 1,622) completed a questionnaire before and after bereavement. The questionnaire assessed “overall functional impairment” (PG-13 item) and “aspects of functional impairment” measured by mean scores of the 36-item Short Form Survey (SF-36) subscales *emotional role* and *social functioning* (0: worst; 100: best). We analyzed associations between PGD_PG13_ and functional impairment prior to bereavement using logistic regression models adjusted for age, gender, personal relation, education, time interval to patient's death, and pre-loss grief.

**Results:** In total, 51% reported overall functional impairment before bereavement, 27% reported functional impairment at 6 months after bereavement, and 19% reported functional impairment at 3 years after bereavement. The mean *emotional role* score was 47.5 (95%CI: 45.4–49.7) before bereavement, increasing to 77.4 (95%CI: 75.7–79.0) at 3 years after bereavement, compared to 85.1 (95%CI: 77.6–92.6) in a reference sample. Mean *social functioning* score increased gradually reaching the mean of the reference sample at 3 years after bereavement. PGD_PG13_ was present in 26% of those with overall functional impairment at 6 months after bereavement, decreasing to 11% at 3 years after bereavement. Pre-bereavement measures of *emotional role* and *social functioning* were associated with PGD_PG13_at 6 months and 3 years after bereavement.

**Discussion:** Overall functional impairment was prevalent as reflected in low scores on daily activities and social functioning compared to a reference sample. Functioning may be an important factor during caregiving and bereavement and pre-bereavement functional impairment was associated with PGD_PG13_.

Future studies should investigate if maintaining daily activities and social functioning before bereavement could be key in early supportive care. Moreover, the role of functional impairment in bereavement interventions should be explored.

## Introduction

Severe illness, bereavement and other losses are likely to impair the ability to maintain daily activities and thereby cause functional impairment (1). The World Health Organization (WHO) defines functional impairment as an umbrella term in the International Classification of Functioning, Disability and Health (ICF) and conceptualizes a biopsychosocial approach to functioning in the context of health (2). Moreover, functional impairment may imply emotional suffering and is a common reason for healthcare seeking (3).

According to the 11th revision of the International Classification of Diseases (ICD-11), prolonged grief disorder (PGD_ICD11_) entails longing for, or preoccupation with, the deceased person, which is accompanied by intense emotional pain, and functional impairment is described as “*impairment of personal, family, social, occupational, educational or other important areas of functioning*” (4). Thus, functional impairment needs to be present to fulfill the diagnostic criteria for PGD_ICD11_ (4).

Functional impairment has been included as a clinical significance criterion in the diagnosis of mental illness in the Diagnostic and Statistical Manual of Mental Disorders (DSM) by the American Psychiatric Association since 1980 (1). It was added as a new criterion in the ICD-11 to improve the distinction between normality and disturbance (5). Hence, including functional impairment in the diagnostic criteria of PGD_ICD11_ may support the clinical utility of the diagnosis, which is intended to help health professionals assess the needs of the bereaved person, to differentiate between PGD and natural grief or other disorders, and to plan treatment (6, 7).

Despite the central role of functional impairment, only few studies have explored aspects of functional impairment in bereaved persons. Maladaptive emotional symptoms of grief have been investigated without including functional impairment; these studies have revealed subgroups of people with different patterns of grief symptoms (8, 9). Functional impairment has been associated with PG symptoms measured on different grief symptom scales in disaster settings (10), in bereaved adolescents (11, 12), and in a small-scale study of bereaved persons recruited from a funeral service (13). A recent cross-sectional study used network analysis to explore associations between aspects of *quality of life impairments* on the WHO abbreviated multidimensional quality-of-life scale (WHOQOL-BREF) and PG symptoms measured on the Prolonged Gried-13 scale (PG-13) (14). The PG-13 includes both grief symptoms and functional impairment. Different aspects of impairments were associated with different PGD_PG13_ symptoms. For example, poor psychological health was associated with the PGD_PG13_ symptoms of *meaninglessness* and *role confusion*, whereas poor social health was associated with the PGD_PG13_ symptom *lack of trust in others*. These results point at functional impairment as a complex concept encompassing a range of aspects that interact with symptoms of PG. However, previous research has focused on functional impairment in bereaved persons suffering from PGD, whereas the role of functional impairment as a risk factor for bereavement outcomes like PGD and depression remains unknown.

Stroebe et al. established groups of risk factors for adverse bereavement outcomes. These risk factors may interact, and they are mediated by the person's coping and emotion regulation (15). Risk factors include *intrapersonal factors*, such as low educational level and a history of poor mental health (16, 17), *interpersonal factors*, such as lack of social support and *situational* factors related to the context of caregiving and death (15). Social network and everyday life, including work are areas related to interpersonal and situational factors. These areas are highlighted in the PGD_ICD11_ criteria. According to the ICF, functional impairment includes bodily functions, activities, and participation in daily life including work and social life, which interact with personal factors and the context (2). The PG-13 scale is widely used for measuring prolonged grief and includes an item of overall functional impairment, without assessment of specific areas (18). The level of impairment in specific areas of daily activities and social functioning can be assessed by the Short Form survey-36 (SF-36) (19).

We aimed to examine the overall extent of functional impairment before bereavement and the level at 6 months after bereavement and at 3 years after bereavement, including the impact on daily activities and social functioning. Furthermore, we aimed to explore the association between emotion-related role and social functioning with PGD_PG13_.

We hypothesized that functional impairment would more often be present in bereaved persons compared to an age-matched reference population, and we anticipated that the extent of functional impairment would decrease to the level seen in a reference sample at 3 years after bereavement when the proportion of severely impaired bereaved persons would be low. Furthermore, we hypothesized that all aspects of functional impairment before bereavement would be associated with PGD_PG13_ after bereavement.

## Methods

### Setting and Design

We conducted a longitudinal, population-based study in a cohort of Danish family caregivers, which was established in 2012. The current study was a secondary analysis. The primary and previous secondary analysis focused on symptoms of grief and depression before and after death (17, 20, 21), trajectories of grief symptoms (8) and physical and mental health (22).

Participants were identified through patients registered with a drug reimbursement scheme due to terminal illness in *the Danish National Database of Reimbursed Prescriptions* (23). On a weekly basis, we sent an information letter and a questionnaire to newly registered patients and requested their closest family caregiver to complete the questionnaire (20). Enrolled family caregivers providing informed consent were asked to complete a questionnaire at inclusion (T0), at 6 months after bereavement (T1), and at 3 years after bereavement (T2).

**Figure 1:**
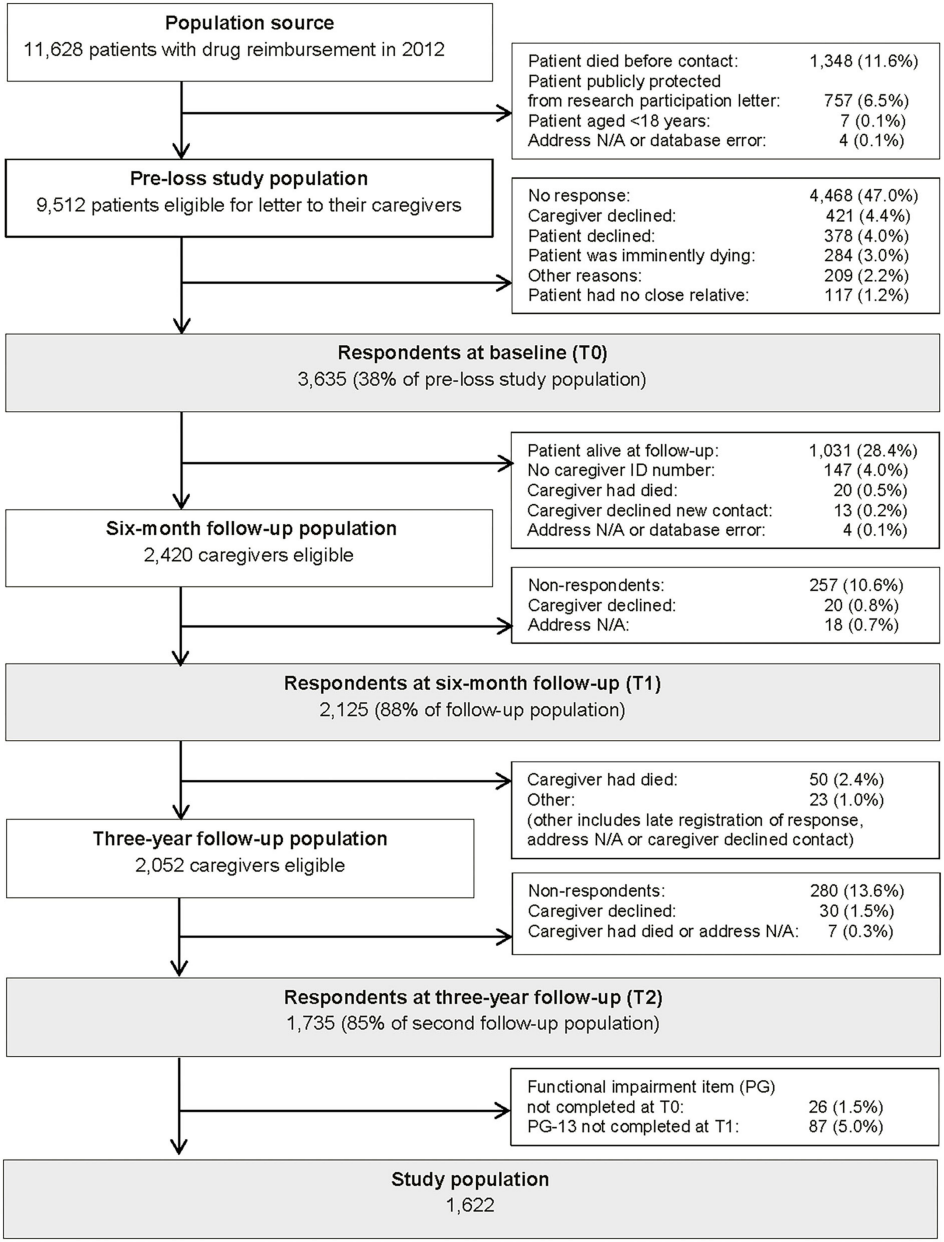
Flow diagram of family caregivers at the time of the patient's drug reimbursement registration due to terminal illness (T0), 6 months after the patient's death (T1), and 3 years after the patient's death (T2).

In this study, we included caregivers who had completed a questionnaire at all three time points, filled in the functional impairment item of the PG-13 scale before bereavement (T0), and the total PG-13 at 6 months after bereavement (T1) (Figure 1).

### Ethics

The Committee on Health Research Ethics of the Central Denmark Region confirmed that this study required no ethical clearance according to the Danish Act on Research Ethics Review of Health Research Projects (24). The study was approved by the Danish Data Protection Agency (file no. 2013-41-2603) and registered in the Record of Processing Activities at the Research Unit for General Practice, Aarhus (Id 207) in accordance with the provisions of the General Data Protection Regulation (GDRP). The participants gave written informed consent to participate in this study.

### Functional Impairment

Overall functional impairment (yes, no) was measured as *social, occupational, and other* impairment in one measure by the item “*Have you experienced a significant reduction in social, occupational, or other important areas of functioning (e.g., domestic responsibilities)?”* of the PG-13. It was reported as proportions with impairment before bereavement, at 6 months after bereavement, and at 3 years after bereavement (18). Functional impairment related to limitations in daily role due to emotional impairment and limitations in social functioning was assessed by the SF-36 subscales *emotional role* and *social functioning* before bereavement, at 6 months after bereavement, and at 3 years after bereavement (19). Subscale mean scores were weighted with scores of the remaining SF-36 subscales according to the manual, and a sum score was derived (25). Scores were graded from zero to 100, with 100 indicating highest performance (i.e., better functioning). Thus, low scores indicate impaired functioning. For comparison, we used standardized mean scores from the general Danish population aged 55–65 years as the mean age of the cohort was 62 years (26).

The *emotional role* subscale consists of three items exploring whether less time is spent on activities (RE1), accomplishment of tasks (RE2), and carefulness devoted to performing activities (RE3) (yes, no). As the items cover functioning in both daily activities and occupation, they are also applicable to retired individuals (19). The *social functioning* subscale consists of two items. One item (SF1) measures whether physical and emotional problems interfere with the extent of social activities with family, friends, neighbors, or others (no (not at all, little), yes (some, a lot, very much)). The other item (SF2) measures the extent of interference from physical health or emotional problems on social activities, such as visiting friends, relatives, or others (not at all, little of the time, some of the time, most of the time, all the time) (19). Raw means of individual items were calculated according to the manual (25).

### Grief, Depressive Symptoms, and Sociodemographic Factors

All items of the PG-13 scale (18) were used to measure fulfillment of the PGD_PG13_ criteria (yes, no) at 6 months and at 3 years after bereavement. To fulfill the criteria, participants must have reported core symptoms of longing/yearning or intense feelings related to the loss at least daily (a score of at least 4 on a 5 point scale), at least five out of nine accessory symptoms such as trouble accepting the loss, bitterness and emotional numbness (a score of at least 4 on a 5 point scale), a duration of 6 months or longer, and functional impairment (18). In collaboration with the author of the PG-13 scale, Dr. Prigerson, the Danish version of the PG-13 was developed according to the WHO recommendation for translation of scales using forward and back ward translation (27). The Cronbach's alpha of the entire scale was 0.90. The documentation of the translation process is described in a paper in preparation.

Grief symptoms during the patient's illness trajectory were assessed by a version of the PGD_PG13_ adapted to terminal illness (20). In the pre-loss version, “the loss” was replaced by “your relative's illness,” the item concerning “moving on” was replaced with an item about concentration problems inspired by a previous study on grief before death (28), and the duration item was left out (please see details in Supplementary Material A).

Depressive symptoms were assessed before bereavement by the Beck Depression Inventory-II (29) and dichotomized according to severity (no-mild, moderate-severe).

Information on the family caregiver's personal relation to the terminally ill patient was obtained at T0 [partner, non-partner (adult child, other relation)]. Data on the family caregiver's age, gender, educational level, and the patient's survival time from drug reimbursement were extracted from the *Danish Civil Registration System* (30).

### Statistical Analysis

Questionnaire data and registry-based data were combined at *Statistics Denmark* (31). Overall functional impairment was reported as proportions. Weighted mean scores of the *emotional role* and *social functioning* subscales and mean scores of the single items (RE1-3 and SF1-2) were reported as means with 95% confidence intervals at T0, T1, and T2. *Emotional role* and *social functioning* mean scores were compared to standard scores in a Danish reference sample aged 55-64 (26), and interpreted using minimal clinical important difference (MCID) between the mean scores of the study sample and the reference sample. The MCID is the smallest change in an outcome score that the individual person will consider important and corresponds to 3–5 units on the SF-36 scale (32).

We used logistic regression with 95% confidence intervals to calculate associations between SF-36 measures of functional impairment before bereavement and PGD_PG13_. We adjusted for age, gender, personal relation, education, time from inclusion to the patient's death, and grief symptom level before death (sum score). These covariates were socioeconomic factors and clinical factors that have been associated with in earlier studies (28, 33). Due to the number of fulfilling the criteria for PGD_PG13_ (*n* = 117 events), inclusion of these covariates did not violate the assumptions of logistic regression (34). Results were reported as odds ratios with 95% confidence intervals. All analyses were conducted by Stata 14 (StataCorp, Texas, USA, RRID:SCR_012763).

## Results

### Study Population

A total of 1,622 bereaved persons were included in the study (Figure 1). The mean age was 62 years (Table 1). Participants were predominantly female (70%), partner to the patient (65%), and 25% had <10 years of education. Furthermore, pre-loss grief (fulfilling the criteria of the adapted PGD_PG13_ before bereavement) was reported in 15 and 16% had depressive symptoms before bereavement. Among those reporting overall functional impairment (PG item), the mean age was lower (60.6 years (59.8; 61.4) vs. 63.0 (62.2; 63.7)) and proportions of females (79% vs. 62%) and participants with depressive symptoms (23% vs. 6%) were higher compared to in participants without overall functional impairment (Table 1).

**Table 1:** Characteristics of the total study cohort (*n* = 1,622).

	**Total cohort** **(*****n*** **=** **1,622)**			Overall functional impairment^*^ **(*****n*** **=** **831)**			**No overall functional impairment** **(*****n*** **=** **791)**		
**Caregiver characteristics**	**Mean (95% CI)**	***n***	**(%)**	**Mean (95% CI)**	***n***	**(%)**	**Mean (95% CI)**	***n***	**(%)**
Age, years^a^	61.8 (61.2; 62.3)			60.6 (59.8; 61.4)			63.0 (62.2; 63.7)		
Gender									
Male		480	(30)		178	(21)		302	(38)
Female		1,142	(70)		653	(79)		489	(62)
Personal relation									
Partner		1,050	(65)		552	(66)		498	(63)
Other		572	(35)		279	(34)		293	(37)
Education									
Low (≤10 years)		405	(25)		204	(25)		201	(25)
Intermediate (>10 and ≤15 years)		780	(48)		388	(47)		392	(50)
High (>15 years)		437	(27)		239	(29)		198	(25)
Pre-death PG criteria^b^									
Not fulfilled criteria		1,345	(83)		570	(69)		775	(98)
Fulfilled criteria		252	(16)		252	(30)		-	-
Missing		25	(2)		9	(1)		16	(2)
Depressive symptoms^c^									
No-mild symptoms		1,318	(81)		601	(72)		717	(91)
Moderate-severe symptoms		239	(15)		195	(23)		44	(6)
Missing		65	(4)		35	(4)		30	(4)
**Patient characteristics**	**Median (IQI)**			**Median (IQI)**			**Median (IQI)**		
Time from drug reimbursement^**^	69.4 (67.0; 71.8)			66.6 (63.4; 69.9)			72.3 (68.7; 75.8)		
to death, days									

* *Based on the impairment item of the PG-13*.

** *Registration of patient with drug reimbursement due to terminal illness and invitation for study inclusion*.

a *Caregivers' age at baseline*.

b *Prolonged grief criteria measured on an adapted pre-death version of the Prolonged Grief-13 scale*.

c *Depressive symptoms measured on Beck's Depression Inventory-II*.

### Functional Impairment Before and After Bereavement

In total, proportions of 51% reported overall functional impairment before bereavement, 27% at 6 months after bereavement, and 19% at 3 years after bereavement measured on the PG-13 impairment item (Table 2). Of those reporting overall functional impairment at 6 months, 63 (54%) also reported overall impairment before bereavement, whereas 21 (68%) of those reporting overall impairment at 3 years also had overall impairment before bereavement.

**Table 2:** Functional impairment in the study sample (*n* = 1,622) before bereavement, at 6 months and 3 years after bereavement and in a standardized reference sample of Danes aged 55–64 years.

	**0–6 months before** **bereavement**		**6 months** **after bereavement**		**3 years after** **bereavement**		**Reference** **sample^§^**		
**Overall functional impairment (PG item)^a^**	***n***	**(%)**	***n***	**(%)**	***n***	**(%)**			
Impairment criterion (single item), yes (%)	831	(51)	438	(27)	306	(19)	NA	NA	
**Emotional role (SF-36)^b^**	**Mean (95% CI)**		**Mean (95% CI)**		**Mean (95% CI)**		**Mean, SD**		
*Emotional role* subscale, weighted mean (95% CI)	47.5	45.4; 49.7	71.5	69.8; 73.3	77.4	75.7; 79.0	85.1	77.6; 92.6	^*^
RE1. Cut down on amount of time spent on work/other activities^c^	1.5	1.5; 1.5	1.78	1.8; 1.8	1.8	1.8; 1.8	1.9	0.29	^#^
RE2. Accomplished less than you would like^c^	1.4	1.4; 1.4	1.55	1.5; 1.6	1.7	1.6; 1.7	1.8	0.40	^#^
RE3. Did not do work or other activities as carefully as usual^c^	1.5	1.5; 1.6	1.8	1.8; 1.8	1.8	1.8; 1.9	1.9	0.30	^#^
**Social functioning (SF-36)**^**b**^					
*Social functioning* subscale, weighted mean (95% CI)	77.3	76.1; 78.5	85.3	84.3; 86.3	88.9	87.9; 89.8	89.9	82.0; 97.8	^*^
SF1. Extent, mean (95% CI)^d^	4.3	4.2; 4.3	4.5	4.4; 4.5	4.6	4.5; 4.6	4.7	0.71	^#^
SF2. Time, mean (95% CI)^e^	3.9	3.9; 4.0	4.4	4.3; 4.4	4.5	4.5; 4.6	4.7	0.74	^#^

a *Functional impairment item of the Prolonged Grief-13 scale (18)*.

b *Short Form 36-item Health Outcome survey (19)*.

c *Text of the RE items: “During the past 4 weeks, have you had any of the following problems with your work or other regular daily activities as a result of any emotional problems (such as feeling depressed or anxious)”*.

d Text of SF1 item: “During the past 4 weeks, to what extent has your physical health or emotional problems interfered with your normal social activities with family, friends, neighbors, or groups?”

e Text of SF2 item: “During the past 4 weeks, how much of the time has your physical health or emotional problems interfered with your social activities (like visiting friends, relatives, etc.)?”

§ *Danish standardized norm population (25)*.

* *Mean (95% CI) in the Danish standardized norm population aged 55–64 years*.

# *Mean (SD) in the total standardized Danish norm population (n = 3,602)*.

*NA: not applicable*.

Before bereavement, the mean *emotional role* score (47.5 (95% CI: 45.4–49.7)) and the mean *social functioning* score (77.3 (95% CI: 76.1–78.5)) were significantly lower compared to the reference sample (85.1 for *emotional role* and 89.9 for *social functioning*) (Table 2). The difference between means was 37.6 for *emotional role* and 12.6 for *social functioning*. As the MCID was three to five for SF-36 subscales, the mean scores were significantly lower in the study sample (The mean scores of both subscales had increased at 6 months after bereavement and almost reached the level of the reference sample at 3 years after bereavement (*emotional role*: 77.4 (95% CI: 75.7.9–79.0), *social functioning*: 88.9 (95% CI: 87.9–89.8)). An MCID of five was reached for the *emotional role* subscale mean score compared to the reference sample, but no MCID was found for the *social functioning* mean score at 3 years after bereavement (Table 2).

The mean scores of all *emotional role* and *social functioning* subscale items were accordingly low in the bereaved persons, and the social functioning item regarding *less time spent on social activity* was very low before bereavement (3.9 (3.9–4.0) compared to a reference sample (4.7 (SD 0.74)) (Table 2).

**Figure 2:**
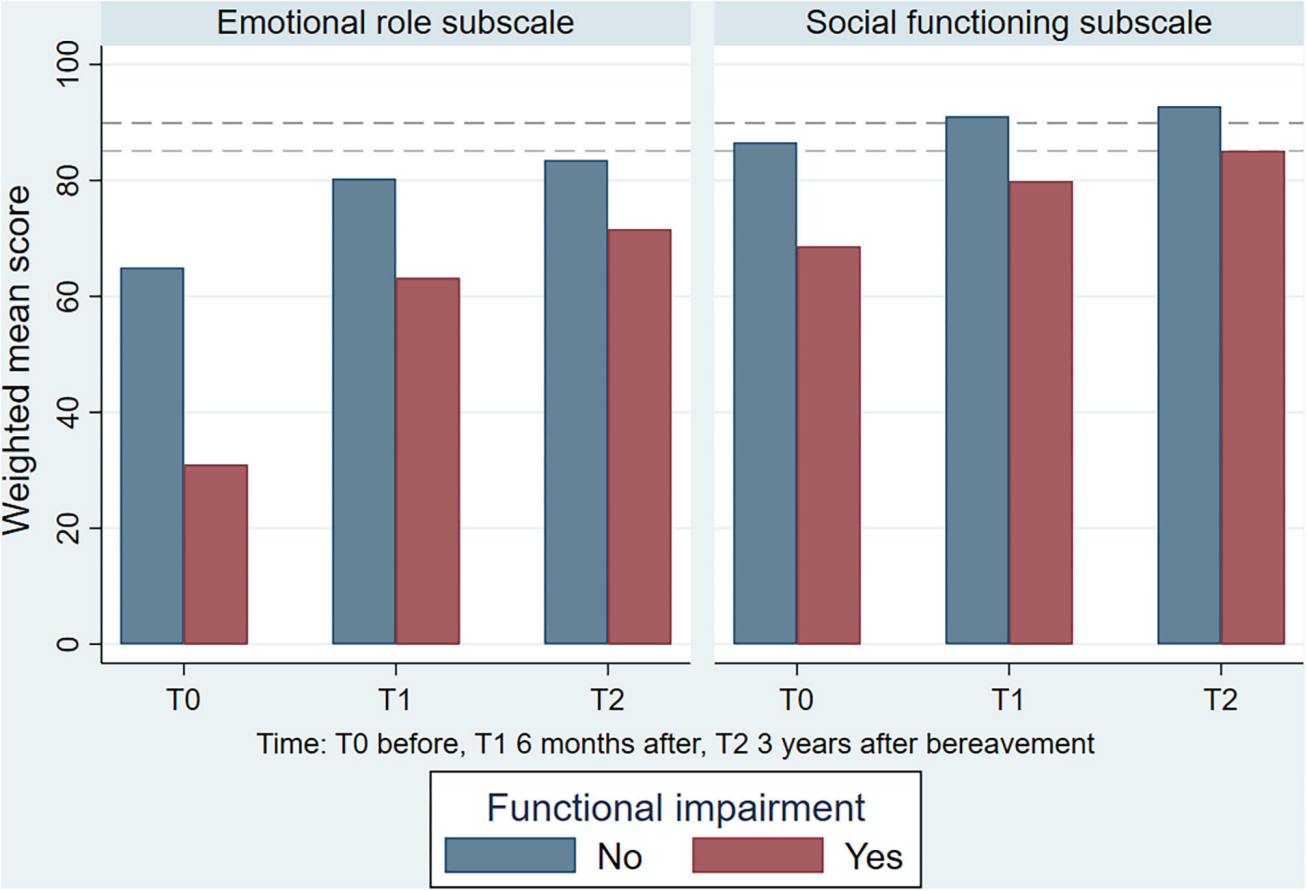
The development in weighted mean scores of the SF-36 subscales *emotional role* and *social functioning* from before to after bereavement according to overall functional impairment (*n* = 1,622). Dashed horizontal lines represents general population sample mean scores.

In those with overall functional impairment before bereavement, the mean scores of *emotional role* (31.0 (95% CI: 28.3–33.7)) and *social functioning* (68.6 (95% CI: 66.8–70.4)) were lower than in the total study cohort before bereavement (Table 3a). Mean scores improved over time (Figure 2). However, an MCID of at least three was present at all measurement points in those with overall functional impairment. Women and those below 60 years of age were more likely to report overall functional impairment and limitation in *social functioning*, whereas women and those above 60 years were more likely to report limitations *emotional role* (Tables 3b,c).

**Table 3a:** Functional impairment aspects (SF-36) before bereavement, 6 months after bereavement, and 3 years after bereavement in the subgroup with overall functional impairment before bereavement (PG item^a^) (51% of the total population).

	**Overall functional impairment^a^ before bereavement (*n* = 851)**					
	**Before bereavement**		**6 months after bereavement**		**3 years after bereavement**	
**Emotional role (SF-36)^b^**						
*Emotional role* subscale, weighted mean (95% CI)	31.0	(28.2; 33.7)	63.2	(60.6; 65.7)	71.6	(69.1; 74.1)
RE1. Cut down on amount of time spent on work/other activities^c^	1.3	(1.3; 1.4)	1.7	(1.7; 1.8)	1.8	(1.7; 1.8)
RE2. Accomplished less than you would like^c^	1.2	(1.2; 1.3)	1.4	(1.4; 1.5)	1.6	(1.6; 1.6)
RE3. Did not do work or other activities as carefully as usual^c^	1.4	(1.3; 1.4)	1.7	(1.7; 1.7)	1.8	(1.8; 1.8)
**Social functioning (SF-36)^b^**						
*Social functioning* subscale, weighted mean (95% CI)	68.6	(66.8; 70.4)	79.8	(78.2; 81.4)	85.1	(83.6; 86.6)
SF1. Extent, mean (95% CI)^d^	3.9	(3.9; 4.0)	4.3	(4.2; 4.3)	4.4	(4.4; 4.5)
SF2. Time, mean (95% CI)^e^	3.5	(3.5; 3.6)	4.1	(4.1; 4.2)	4.4	(4.3; 4.5)

a *Functional impairment item of the Prolonged Grief-13 scale (18)*.

b *Short Form-36 Health Outcome survey (19)*.

c *Text of the RE items: “During the past 4 weeks, have you had any of the following problems with your work or other regular daily activities as a result of any emotional problems (such as feeling depressed or anxious)”*.

d Text of the SF1 item: “During the past 4 weeks, to what extent has your physical health or emotional problems interfered with your normal social activities with family, friends, neighbors, or groups?”

e Text of the SF2 item: “During the past 4 weeks, how much of the time has your physical health or emotional problems interfered with your social activities (like visiting friends, relatives, etc)?”

**Table 3b:** Functional impairment aspects (SF-36) before bereavement, 6 months after bereavement, and 3 years after bereavement according to gender.

	**Functional impairment, males (*****n*** **=** **480 (30%))**					
	**Before bereavement**		**6 months after bereavement**		**3 years after bereavement**	
**Overall functional impairment (PG-13 scale)^a^**						
Yes, *n* (%)	178	(37)	95	(20)	75	(16)
**Aspects of functional impairment (SF-36 scale)^b^**						
*Emotional role* subscale, weighted mean (95% CI)	53.4	(49.5; 57.4)	74.0	(70.9; 77.1)	76.6	(73.5; 79.6)
*Social functioning* subscale^2^, weighted mean (95% CI)	82.8	(80.8; 84.8)	89.2	(87.5; 90.9)	89.4	(87.7; 91.2)
	**Functional impairment, females (*****n*** **=** **1,142 (70%))**					
	**Before bereavement**		**6 months after bereavement**		**3 years after bereavement**	
**Overall functional impairment (PG-13 scale)^a^**						
Yes, *n* (%)	653	(57)	343	(30)	213	(20)
**Aspects of functional impairment (SF-36 scale)^b^**						
*Emotional role* subscale, weighted mean (95% CI)	45.0	(42.5; 47.6)	70.5	(68.4; 72.6)	77.7	(75.7; 79.7)
*Social functioning* subscale^b^, weighted mean (95% CI)	75.1	(73.6; 76.5)	83.7	(82.4; 85.0)	88.6	(87.5; 89.8)

a *Functional impairment item of the Prolonged Grief-13 scale (18)*.

b *Short Form-36 Health Outcome survey (19)*.

**Table 3c:** Functional impairment aspects (SF-36) before bereavement, 6 months after bereavement, and 3 years after bereavement according to age.

	**Age** ** <60 (*****n*** **=** **665 (41%))**					
	**Before bereavement**		**6 months after bereavement**		**3 years after bereavement**	
**Overall functional impairment (PG-13 scale)^a^**						
Yes, *n* (%)	384	(58)	210	(32)	137	(21)
**Aspects of functional impairment (SF-36 scale)^*b*^**						
Emotional role subscale, weighted mean (95% CI)	48.4	(45.1; 51.6)	72.6	(69.9; 75.3)	79.7	(77.2; 82.3)
Social functioning subscale^*b*^, weighted mean (95% CI)	76.3	(74.4; 78.2)	83.1	(81.4; 84.8)	87.8	(86.2; 89.4)
	**Age** **>60 (*****n*** **=** **957 (59%))**					
	**Before bereavement**		**6 months after bereavement**		**3 years after bereavement**	
**Overall functional impairment (PG-13 scale)^a^**						
Yes, *n* (%)	447	(47)	228	(24)	169	(18)
**Aspects of functional impairment (SF-36 scale)^b^**						
Emotional role subscale, weighted mean (95% CI)	47.0	(44.1; 49.8)	70.7	(68.5; 73.0)	75.7	(73.5; 79.0)
Social functioning subscale^*b*^, weighted mean (95% CI)	78.1	(76.5; 79.6)	86.8	(85.5; 88.1)	89.6	(88.4; 90.8)

a *Functional impairment item of the Prolonged Grief-13 scale (18)*.

b *Short Form-36 Health Outcome survey (19)*.

Of the 438 bereaved persons reporting overall functional impairment at 6 months after bereavement, 27% fulfilled the PGD_PG13_ criteria. Accordingly, 10% of those reporting overall functional impairment at 3 years after bereavement fulfilled the PGD_PG13_ criteria (data not shown).

### Functional Impairment Before Bereavement and PGD_PG13_

Functional impairment before bereavement was associated with PGD_PG13_ at 6 months after bereavement on the *emotional role* (OR = 0.97 (95% CI 0.96; 0.98)) and *social functioning* (OR = 0.97 (95% CI 0.96; 0.98)) subscales of the SF-36 (Tables 4 and 5). The *emotional role* items indicating *less time spent on work or activities, accomplishment of tasks*, and *carefulness in performing activities*, and both *social functioning* items indicating *lower extent* and *less time spent on social network*, were also associated with PGD_PG13_.

**Table 4:** Functional impairment (SF-36 subscales) and the association with prolonged grief disorder (PG-13)^a^ at 6 months after bereavement in the total cohort (*N* = 1,622) and in the subgroup with overall functional impairment before bereavement (*n* = 831)^b^.

	**PG-13 Prolonged grief disorder at 6 months after bereavement^a^**							
	**Total cohort**				**Overall functional impairment^b^ before bereavement**			
	**No PG (*****n*** **=** **1,505)**		**PG (*****n*** **=** **117)**		**No (*****n*** **=** **742 (89%))**		**Yes (*****n*** **=** **89 (11%))**	
**Emotional role (SF-36)^c^**								
*Emotional role* subscale, weighted mean (95% CI)	50.0	(47.7; 52.2)	16.4	(11.0; 21.8)	33.0	(30.1; 35.9)	13.8	(8.1; 19.5)
RE1. Cut down on amount of time spent on work/other activities^d^	1.5	(1.5; 1.5)	1.2	(1.2; 1.3)	1.4	(1.3; 1.4)	1.2	(1.1; 1.3)
RE2. Accomplished less than you would like^d^	1.4	(1.4; 1.4)	1.1	(1.0; 1.2)	1.3	(1.2; 1.3)	1.1	(1.0; 1.1)
RE3. Did not do work or other activities as carefully as usual^d^	1.6	(1.5; 1.6)	1.2	(1.1; 1.2)	1.4	(1.3; 1.4)	1.1	(1.1; 1.2)
**Social functioning (SF-36)^c^**								
*Social functioning* subscale, weighted mean (95% CI)	79.0	(77.8; 80.2)	56.4	(51.8; 61.0)	70.4	(68.6; 72.3)	53.0	(48.0; 58.0)
SF1. Extent, mean (95% CI)^e^	4.3	(4.3; 4.4)	3.5	(3.3; 3.7)	4.0	(3.9; 4.1)	3.4	(3.1; 3.6)
SF2. Time, mean (95% CI)^f^	4.0	(3.9; 4.0)	3.0	(2.8; 3.2)	3.6	(3.5; 3.7)	2.9	(2.7; 3.1)

a *Prolonged Grief-13 scale (18)*.

b *Functional impairment item of the Prolonged Grief-13 scale (18)*.

c *Short Form 36-item Health Outcome survey (19)*.

d *Text of the RE items: “During the past 4 weeks, have you had any of the following problems with your work or other regular daily activities as a result of any emotional problems (such as feeling depressed or anxious)”*.

e Text of SF1 item: “During the past 4 weeks, to what extent has your physical health or emotional problems interfered with your normal social activities with family, friends, neighbors, or groups?”

f Text of SF2 item: “During the past 4 weeks, how much of the time has your physical health or emotional problems interfered with your social activities (like visiting friends, relatives, etc.)?”

**Table 5:** Functional impairment before bereavement and associations with prolonged grief disorder (PG-13)^a^ at 6 months and at 3 years after bereavement (*n* = 1,622).

	**Prolonged grief disorder (PG-13)**		
	**6 months after bereavement**		**3 years after bereavement**
	**Unadjusted OR (95% CI)**	**Adjusted^#^ OR (95% CI)**	**Unadjusted OR (95% CI)**
**Emotional role (SF-36)^b^**			
*Emotional role* subscale, weighted mean (95% CI)	0.98 (0.97; 0.98)	0.98 (0.98; 0.99)	0.98 (0.97; 0.99)
RE1. Cut down on amount of time spent on work/other activities^c^	0.27 (0.17; 0.43)	0.43 (0.27; 0.70)	0.347 (0.15; 0.76)
RE2. Accomplished less than you would like^c^	0.15 (0.08; 0.28)	0.24 (0.13; 0.46)	0.16 (0.05; 0.53)
RE3. Did not do work or other activities as carefully as usual^c^	0.16 (0.10; 0.26)	0.25 (0.15; 0.43)	0.13 (0.04; 0.37)
**Social functioning (SF-36)^b^**			
*Social functioning* subscale, weighted mean (95% CI)	0.97 (0.96; 0.98)	0.98 (0.97; 0.98)	0.97 (0.96; 0.98)
SF1. Extent, mean (95% CI)^d^	0.52 (0.45; 0.61)	0.63 (0.53; 0.76)	0.53 (0.40; 0.70)
SF2. Time, mean (95% CI)^e^	0.54 (0.47; 0.63)	0.63 (0.53; 0.75)	0.52 (0.39; 0.68)

# *Logistic regression model adjusted for age, gender, personal relation to the patient, education, time from inclusion to the patient's death and grief symptoms before death*.

a *Prolonged Grief-13 scale (18)*.

b *Short Form 36-item Health Outcome survey (19)*.

c *Text of the RE1-3 items: “During the past 4 weeks, have you had any of the following problems with your work or other regular daily activities as a result of any emotional problems (such as feeling depressed or anxious)”*.

d Text of SF1 item: “During the past 4 weeks, to what extent has your physical health or emotional problems interfered with your normal social activities with family, friends, neighbors, or groups?”

e Text of SF2 item: “During the past 4 weeks, how much of the time has your physical health or emotional problems interfered with your social activities (like visiting friends, relatives, etc.)?”

Functional impairment before bereavement on the *emotional role* and *social functioning* subscales was also associated with PGD_PG13_ at 3 years after bereavement in unadjusted analysis (Table 5).

## Discussion

### Key Findings

The present study explored functional impairment in family caregivers and revealed novel findings. Firstly, functional impairment was present in a large proportion of bereaved persons before the bereavement, and the scores relating to specific aspects of impairment in daily activities and social functioning were much lower in this group compared to a reference sample. Secondly, the functional impairment improved over time. The mean *social functioning* scores reached the level of the reference sample at 3 years after bereavement, whereas the mean *emotional role* scores remained significantly lower than in the reference sample at 3 years after bereavement. In the subgroup with overall functional impairment before bereavement, the *emotional role* and *social functioning* mean scores were even lower than in the total cohort.

Thirdly, low scores on the *emotional role* and *social functioning* subscales and specific aspects of reduced time, accomplishment, carefulness in performing activities, and extent and time spent on social activities before bereavement were risk factors for PGD_PG13_.

### Comparison With Previous Finding

The extent of functional impairment was surprisingly high at all measurement points. We used the PG item of functional impairment to measure overall impairment. This broad measure included social, occupational, and other important areas of functioning and is likely to capture functioning in general. By investigating functional impairment aspects on the SF-36 *emotional role* and *social functioning* subscales, we showed that limited “daily role functioning due to emotional state” and reduced “social functioning” were associated with overall functional impairment. This is in line with our hypothesis. The ICF interprets functional impairment in a biopsychosocial framework, emphasizing the importance of maintaining daily activities in the context of bereavement (2). Our findings of high overall functional impairment support the interpretation of functional impairment according to the ICF. Moreover, we established new knowledge as we found that a high proportion of relatives were functionally impaired; this knowledge can support the clinical assessment of bereaved persons and enhance the planning of health care services before and after bereavement.

Half of the participants reported overall functional impairment before the bereavement. End-of-life care is highly demanding for relatives, and the situation may affect their quality of life and cause psychological distress (35). Our study did not investigate the functional impairment before the end-of-life period and did not include a matched comparison group. This limits our opportunity to conclude that the functional impairment was caused by caregiving. Nevertheless, the very low mean scores of *emotional role* and *social functioning* compared to the reference sample suggest that end-of-life care considerably affects the functioning of relatives. The impact of caregiving has been explored in a comparative study (*n* = 70); this study showed that grief symptoms were more present and that general health and quality of life were significantly worse in family caregivers than in the comparison group (36). The measures of grief, general health and quality of life improved 9–10 months after bereavement. We found similar results in a large-scale sample, although functional impairment in terms of emotional impact on everyday role was still shown at 3 years after bereavement.

We showed a pattern of improved functioning in the period from before to after bereavement. This finding is in line with earlier research based on the present cohort, which showed that a higher proportion of bereaved individuals reported psychological distress (i.e., symptoms of grief and depression) before bereavement than after bereavement (17). Still, a high proportion (19%) reported overall functional impairment at 3 years after bereavement, which was unexpected as we hypothesized that only a few would still be functionally impaired for a long time after bereavement. Of the bereaved persons with overall functional impairment at 3 years after bereavement, only a small proportion fulfilled the criteria of PGD_PG13_. Previous studies have focused on the functional impairment in bereaved persons as a consequence of PGD (10–12) and used the level of functional impairment to monitor the status of the condition. However, our findings establish that functional impairment in daily activities, social network and other areas plays an important role in the grief process in general, not only in bereaved persons with severe bereavement reactions like PGD.

In a previous study, we found that poor mental and physical health status measured by the SF-36, including the subscales of *emotional role* and *social functioning*, were risk factors for PGD_PG13_. Importantly, we now provide detailed knowledge on the aspects associated with functional impairment. Aspects of daily activities, including time spent, accomplishment, and carefulness in performing activities, but also time and extent of social activities were severely affected before bereavement in our cohort compared to the general population sample, and these aspects constituted risk factors of PGD_PG13_. Daily activities prior to bereavement are related to the caregiver context and constitute situational factors in the *integrated risk factor framework for bereavement outcome*. The authors of the framework describes caregiver burden residue as a restoration-oriented stressor that may affect the adaptation to the bereavement (15). A few previous studies point to caregiver burden as a risk factor for PGD (15, 37). However, we did not find such association in a prior study, which was based on the cohort of the current study, when using the Burden Scale for Family Caregivers as a measurement tool (17). Nevertheless, we now show that impairment of specific areas of the relative's daily activities and social life during caregiving increases the risk of developing PGD_PG13_.

Reduced extent and time spent on social activities during end-of-life care was also associated with PGD_PG13_. Lack of social support has been associated with adverse bereavement outcome, but it is not well-established as a bereavement-specific risk factor (15). In a recent cross-sectional study based on network analysis, Maccallum et al. showed that social functioning (measured by the WHO Quality of Life scale) was associated with the PGD_PG13_ item *lack of trust in other* (14). The cross-sectional design did not allow for causal inferencoe in the network analysis, whereas the present findings point at reduced social activities prior to bereavement as an *interpersonal* risk factor for PGD_PG13_.

However, both a lack of social network before the patient's illness trajectory and reduced social activities due to end-of-life care may affect social life before bereavement and contribute to the development of PGD. The present study lacks information on social network before the illness trajectory as a contributing factor. Yet, the low *social functioning* scores in the study sample compared to the reference sample indicate that reduced activity because of affected physical and mental health in the end-of-life period is an important factor.

### Strengths and Limitations

The strengths of the study are the population-based and prospective study design, the use of validated scales (SF-36 and PG-13), and the use of Danish registers with low levels of missing data. Additional advantages were the large sample size and the systematically collected data, although some selection bias might have been present. We contacted patients who had recently been registered under the public drug reimbursement scheme due to terminal illness, and we received completed questionnaires from relatives to 38% of these patients. At baseline, the patients of responding relatives were higher educated than were non-respondents, and relatives responding at follow-up were better educated than were non-respondents. Since high education has been associated with reduced risk of PGD_PG13_ (17), the participants included in the present study might have had better average mental health, and the extent of PGD_PG13_could thus have been underestimated. Additionally, we expected that functional impairment would reduce the participation in the study. As the present study sample is likely to have had better functioning than the average population of relatives, the level of functional impairment in the present study might have been underestimated. However, we believe that selection bias is unlikely to have affected the association found between functional impairment and PGD_PG13_.

No comparison sample was included in this cohort study and this limits direct comparison with non-bereaved individuals. Hence, it was not possible to conclude on the effect of caregiving on functional impairment. Information on patients' symptom burden was not available, which may account for some of the residual confounding of the analysis as symptom burden is likely to impact the function of the participants and could also be associated with PGD. A clinical interview is necessary to diagnose PGD and the use of self-report questionnaires in this study may overestimate symptom levels (38). However, the use of self-report questionnaires enables the large-scale data collection in a general population caregiver cohort. Another shortcoming was that the measure of overall functional impairment (the PG functional impairment item) was a PG-13 item; this limited our opportunity to analyze the associations between overall functional impairment and PGD_PG13_. Furthermore, we included no specific measure of occupational impairment. Still, the impact on daily activities may better cover the situation of all bereaved persons because loss and bereavement become increasingly common with higher age, and a large number of bereaved persons is likely to have retired. In the present study cohort, up to half of the participants had retired before the bereavement (20). Nevertheless, future studies should investigate the impact of bereavement on occupation in the group of bereaved persons in the work force.

We used the PG impairment item to assess overall functional impairment. This measure is broad and includes both occupational, social, and other impairment in a single item. Still, the PG impairment item may not fully capture ICD-11 functional impairment, which also includes impairment regarding personal, family, and educational functioning. Thus, the used measure could have underestimated the level of functional impairment. Furthermore, the PGD_PG13_ scale was developed in 2009 before the inclusion of PGD_ICD11_ as a diagnosis in 2018. Hence, the PGD_PG13_ is not a diagnostic tool and it does not fully account for the PGD_ICD11_ (38). The core symptom of intense yearning and half of the symptoms of intense emotional pain highlighted in the PGD_ICD11_ and functional impairment are covered using the PGD_PG13_. Still, the PGD_PG13_ was found to identify 59% of clinical cases verified in a Structured Clinical Interview for Complicated Grief (SCI-CG), whereas 95.8% was identified using the PGD_ICD11_ (39). This may limit the generalizability of our results to clinical care. However, the association between functional impairment and PGD_PG13_ is independent of the differences between PGD_PG13_ and PGD_ICD11_. Thus, our findings that impaired daily functioning due to emotional problems and impaired social functioning is associated with developing high levels of grief symptoms still shed light on areas of interest for clinical interventions during caregiving. In line with prior studies based on the study sample (17, 20), the findings may be generalized to populations of family caregivers in similar health care settings.

### Clinical Implications

Temporary functional impairment due to grief may improve over time in most persons as the grief reactions gradually lessen. Nevertheless, several bereaved persons in this sample had functional impairment for years after bereavement. Hence, healthcare interventions to restore the daily functioning may be necessary for some groups. Preventive interventions from health professionals before bereavement should focus on maintaining the daily functioning and social networks, which could also benefit the severely ill patient.

Functional impairment is a frequent reason for seeking healthcare (3), and health professionals should investigate for underlying mental illness. Prior studies have shown that PGD_PG13_ (40), persistently high grief symptom levels (8, 41), depressive symptoms (42), self-harm, suicide, and psychiatric illness (43) were long-term adverse bereavement outcomes. Since functional impairment before bereavement was associated with PGD_PG13_, health professionals need to direct attention toward these persons at an early time point during caregiving to help them balance risk factors and prevent PGD development.

The new knowledge gained from the current study may support the clinical usefulness of functional impairment in the PGD_ICD11_ criteria. However, the PGD_ICD11_ do not include specific criteria to assess functional impairment; they rely on a thorough clinical assessment by a physician. Clinicians have recommended such flexibility to be able to consider PGD in bereaved persons maintaining a high level of functioning. Thus, assessment of both symptoms and daily functioning is crucial in the clinical care for bereaved persons.

### Future Research

Future studies need to investigate the role of functional impairment after bereavement, including occupational impairment such as sick leave due to bereavement and impairment in bodily function. Studies are called for to explore whether bereaved persons would benefit from early supportive intervention to maintain their daily functioning and their social network already before bereavement. Such studies should also address the crucial question: Could development of PGD be prevented by promoting a better balance in daily life?

## Conclusion

The present study establishes functional impairment as an important risk factor in bereavement and functional impairment was common both before and after bereavement. Although functional impairment was reduced over time, the loss may affect the daily activities of bereaved persons for a long time. Impairment in both daily work/activity aspects and social aspects before bereavement was associated with the development of PGD_PG13_. Hence, bereaved persons may benefit from early supportive intervention to maintain their daily functioning and social network already before bereavement.

Large groups with functional impairment were identified at 6 months and 3 years after bereavement, but only small proportions had PGD_PG13_. Hence, functional impairment call for clinical assessment of mental well-being, as it may be related to development of conditions such as depression, or it could be an independent long-term consequence of bereavement. Future research is needed to further establish the role of functional impairment in bereavement, including impairment in bereaved persons in the work force and potential associations with other adverse bereavement outcomes.

## Data Availability Statement

The datasets generated for this study will not be made publicly available. Data sharing is not possible due to protection of personal information.

## Ethics Statement

The Committee on Health Research Ethics of the Central Denmark Region confirmed that this study required no ethical clearance according to the Danish Act on Research Ethics Review of Health Research Projects (24). The study was approved by the Danish Data Protection Agency (file no. 2013-41-2603) and registered in the Record of Processing Activities at the Research Unit for General Practice, Aarhus (Id 207) in accordance with the provisions of the General Data Protection Regulation (GDRP). The participants gave written informed consent to participate in this study.

## Author Contributions

MNi, KC, MNe, PB, and M-BG designed the study. MNi undertook the statistical analysis, wrote the first draft of the manuscript, and M-BG commented on the first draft. All authors contributed to the revised version and approved the final manuscript.

## Conflict of Interest

The authors declare that the research was conducted in the absence of any commercial or financial relationships that could be construed as a potential conflict of interest.
