# Supplementary material for: Exploring Functional Impairment in Light of Prolonged Grief Disorder: A Prospective, Population-Based Cohort Study
Source: Front Psychiatry. 2020 Dec 9;11:537674. doi: 10.3389/fpsyt.2020.537674 (PMC7755858; doi:10.3389/fpsyt.2020.537674)
Supplement: Supplementary file 1 [file Data_Sheet_1.docx]

**Supplementary material A.** Pre-death grief items based on the PG-13 scale by Prigerson et al.

During the past month:

Separation Distress: (#1 or 2 at least daily)

- - - #1: How often have you felt yourself longing or yearning for the time before your relative’s illness?
    - #2: How often have you had intense feelings of emotional pain, sorrow, or pangs of grief related to your relative’s illness?

Cognitive, Emotional and Behavioral Symptoms:

(at least 5 items #3-4: once a day or #5-11: quite a bit )

- - - #3: How often have you tried to avoid reminders that your relative is seriously ill?
    - #4: How often have you felt stunned, shocked, or dazed by your relative’s illness?
    - #5: Do you feel confused about your role in life or feel like you don’t know who you are (i.e., feeling that a part of yourself has died)?

Cognitive, Emotional and Behavioral Symptoms:

(at least 5 items #3-4: once a day or #5-11: quite a bit )

- - - #6: Have you had trouble accepting your relative’s illness?
    - #7: Has it been hard for you to trust others since your relative became ill?
    - #8: Do you feel bitter over your relative’s illness?
    - #9: Do you think about your relative’s illness so much that it is hard to concentrate on anything else or do the things you normally do?^2^
    - #10 Do you feel emotionally numb since your relative became ill?
    - #11: Do you feel that life is unfulfilling, empty, or meaningless since your relative became ill?

Impairment Criterion: (yes)

- - - #12: Have you experienced a significant reduction in social, occupational, or other important areas of functioning (e.g., domestic responsibilities)?
